# (2,9-Dimethyl-4,7-diphenyl-1,10-phen­anthroline-κ^2^ *N*,*N*′)bis­(thio­cyanato-κ*S*)mercury(II)

**DOI:** 10.1107/S1600536809023228

**Published:** 2009-06-24

**Authors:** Robabeh Alizadeh

**Affiliations:** a Damghan University of Basic Sciences, School of Chemistry, Damghan, Iran

## Abstract

In the mol­ecule of the title compound, [Hg(NCS)_2_(C_26_H_20_N_2_)], the Hg^II^ atom is four-coordinated in a distorted tetra­hedral configuration by two N atoms from a chelating 2,9-dimethyl-4,7-diphenyl-1,10-phenanthroline ligand and by two S atoms from two thio­cyanate anions. The ligand ring system is not planar. The dihedral angle  between the phenyl rings is 53.20 (3)° . In the crystal structure, π–π contacts between phenanthroline rings [centroid–centroid distance = 3.981 (1) Å] may stabilize the structure.

## Related literature

For related structures, see: Ahmadi *et al.* (2008 ▶); Alizadeh *et al.* (2009 ▶); Hughes *et al.* (1985 ▶); Kalateh *et al.* (2008 ▶); Khoshtarkib *et al.* (2009 ▶); Mahjoub & Morsali (2003 ▶); Morsali (2006 ▶); Morsali *et al.* (2003 ▶, 2004 ▶); Safari *et al.* (2009 ▶); Tadayon Pour *et al.* (2008 ▶); Xie *et al.* (2004 ▶); Yousefi *et al.* (2009 ▶); Yousefi, Rashidi Vahid *et al.* (2008 ▶); Yousefi, Tadayon Pour *et al.* (2008 ▶). For bond-length data, see: Allen *et al.* (1987 ▶).
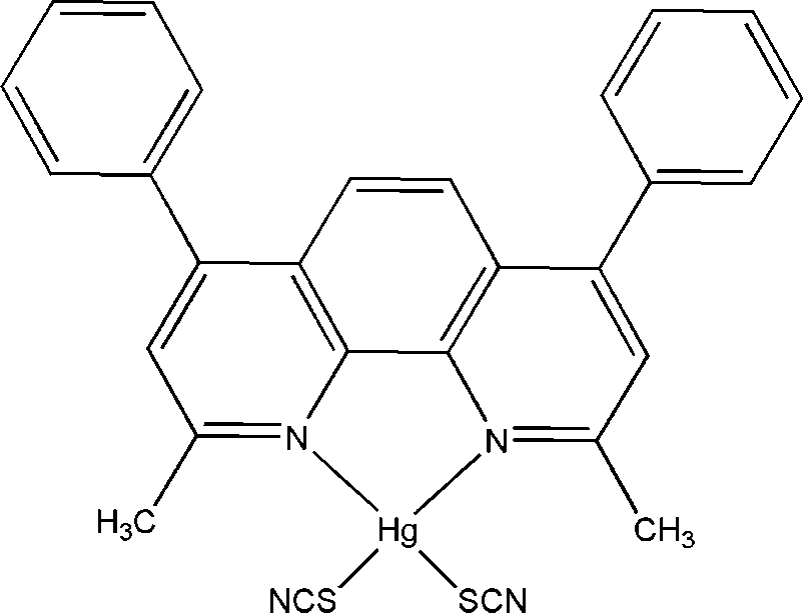


## Experimental

### 

#### Crystal data

[Hg(NCS)_2_(C_26_H_20_N_2_)]
*M* *_r_* = 677.21
Orthorhombic, 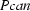

*a* = 7.5907 (3) Å
*b* = 24.0254 (10) Å
*c* = 28.5284 (14) Å
*V* = 5202.7 (4) Å^3^
*Z* = 8
Mo *K*α radiation
μ = 6.10 mm^−1^
*T* = 298 K
0.40 × 0.05 × 0.04 mm

#### Data collection

Bruker SMART CCD area-detector diffractometer
Absorption correction: multi-scan (*SADABS*; Bruker, 1998 ▶) *T*_min_ = 0.711, *T*_max_ = 0.789
56419 measured reflections
7051 independent reflections
4018 reflections with *I* > 2σ(*I*)
*R*_int_ = 0.091

#### Refinement

*R*[*F* ^2^ > 2σ(*F* ^2^)] = 0.093
*wR*(*F* ^2^) = 0.199
*S* = 1.21
7051 reflections
318 parameters
H-atom parameters constrained
Δρ_max_ = 2.55 e Å^−3^
Δρ_min_ = −1.43 e Å^−3^

### 

Data collection: *SMART* (Bruker, 1998 ▶); cell refinement: *SAINT* (Bruker, 1998 ▶); data reduction: *SAINT*; program(s) used to solve structure: *SHELXTL* (Sheldrick, 2008 ▶); program(s) used to refine structure: *SHELXTL*; molecular graphics: *ORTEP-3 for Windows* (Farrugia, 1997 ▶); software used to prepare material for publication: *WinGX* (Farrugia, 1999 ▶).

## Supplementary Material

Crystal structure: contains datablocks I, global. DOI: 10.1107/S1600536809023228/hk2711sup1.cif

Structure factors: contains datablocks I. DOI: 10.1107/S1600536809023228/hk2711Isup2.hkl

Additional supplementary materials:  crystallographic information; 3D view; checkCIF report

## Figures and Tables

**Table:** 

Hg1—N2	2.309 (10)
Hg1—N1	2.320 (10)
Hg1—S2	2.443 (3)
Hg1—S1	2.456 (4)

**Table:** 

N2—Hg1—N1	71.7 (3)
N2—Hg1—S2	115.6 (2)
N1—Hg1—S2	118.6 (2)
N2—Hg1—S1	119.1 (2)
N1—Hg1—S1	114.7 (3)
S2—Hg1—S1	112.00 (12)

**Fig. 1.**
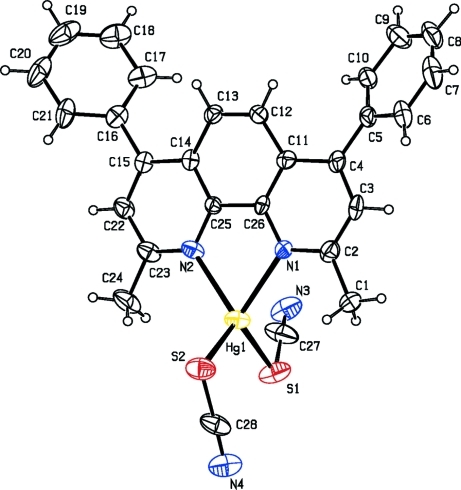
The molecular structure of the title molecule, with the atom-numbering scheme. Displacement ellipsoids are drawn at the 30% probability level.

**Fig. 2.**
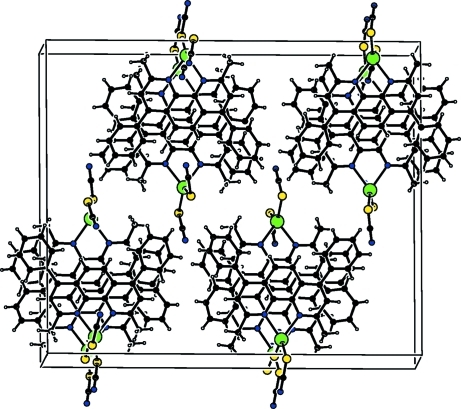
A partial packing diagram of the title compound.

## supplementary crystallographic information

### Comment

Recently, we reported the synthes and crystal structures of [Zn(phend)Cl_2_], (II), (Alizadeh *et al.*, 2009) and [Hg(2,9-dmphen)Br_2_], (III), (Khoshtarkib *et al.*, 2009) [where phend is phenanthridine and 2,9-dmphen is 2,9-dimethyl-1,10-phenanthroline]. There are several Hg^II^ complexes, with formula, [Hg(N—N)*X*_2_], (*X*=Br, Cl, I and SCN), such as [Hg(TPA)Br_2_], (IV), (Xie *et al.*, 2004), [Hg(TPD)Br_2_], (V), (Hughes *et al.*, 1985), [Hg(NH(py)_2_)Br_2_], (VI), (Kalateh *et al.*, 2008), [Hg(6-mbpy)Cl_2_], (VII), (Ahmadi *et al.*, 2008), [Hg(NH(py)_2_)Cl_2_], (IIX), (Yousefi, Allahgholi Ghasri *et al.*, 2009), [Hg(4,4'-dmbpy)I_2_], (IX), (Yousefi, Tadayon Pour *et al.*, 2008), [Hg(5,5'-dmbpy)I_2_], (*X*), (Tadayon Pour *et al.*, 2008), [Hg(ph_2_phen)I_2_], (XI), (Yousefi, Rashidi Vahid *et al.*, 2008), [Hg(SCN)_2_(TBI)], (XII), (Morsali 2006), [Hg(dp4bt)(SCN)_2_], (XIII), (Mahjoub & Morsali 2003), [Hg(da4bt)(SCN)_2_], (XIV), (Morsali *et al.*, 2003), [Hg(biq)(SCN)_2_].C_6_H_6_, (XV), (Morsali *et al.*, 2004) and [Hg(dm4bt)(SCN)_2_], (XVI), (Safari *et al.*, 2009) [where TPA is tris(2-pyridyl)amine, TPD is *N*,*N*,*N*',*N*'-Tetramethyl-*o*-phenylenediamine, NH(py)_2_ is di-2-pyridylamine, 6-mbpy is 6-methyl-2,2'-bipyridine, 4,4'-dmbpy is 4,4'-dimethyl-2,2'-bipyridine, 5,5'-dmbpy is 5,5'-dimethyl-2,2'-bipyridine, ph_2_phen is 4,7-diphenyl-1,10-phenanthroline, TBI is 4,4',5,5'-tetramethyl-2,2'-bi-imidazole, dp4bt is 2,2'-diphenyl-4,4'-bithiazole, da4bt is 2,2'-diamino-4,4'-bithiazole, biq is 2,2'-biquinoline and dm4bt is 2,2'-dimethyl-4,4'-bithiazole] have been synthesized and characterized by single-crystal X-ray diffraction methods. We report herein the synthesis and crystal structure of the title compound (I).

In the molecule of the title compound (Fig 1), Hg^II^ atom is four-coordinated in a distorted tetrahedral configuration by two N atoms from 2,9-dimethyl-4,7-diphenyl-1,10-phenanthroline and two S atoms from two thiocyanate anions (Table 1). The bond lengths (Allen *et al.*, 1987) and angles are within normal ranges, and are in accordance with the corresponding values in (XV). Rings A (N1/C2-C4/C11/C26), B (C11-C14/C25/C26) and C (N2/C14/C15/C22/C23/C25) are, of course, planar and the dihedral angles between them are A/B = 5.43 (3), A/C = 6.53 (3) and B/C = 4.07 (3) °. So, the phenanthroline ring system is not planar. The phenyl rings D (C5-C10) and E (C16-C21) are oriented at a dihedral angle of 53.20 (3)°.

In the crystal structure (Fig. 2), the π–π contact between the phenanthroline rings, Cg2—Cg2^i^ [symmetry code: (i) 1/2 + x, 1/2 - y, z, where Cg2 is centroid of the ring B (C11-C14/C25/C26)] may stabilize the structure, with centroid-centroid distance of 3.981 (1) Å.

### Experimental

For the preparation of the title compound, (I), a solution of 2,9-dimethyl-4,7-diphenyl-1,10-phenanthroline (0.36 g, 1.10 mmol) in HCCl_3_ (20 ml) was added to a solution of Hg(SCN)_2_ (0.35 g, 1.10 mmol) in methanol (20 ml) and the resulting pale yellow solution was stirred for 20 min at room temperature, and then it was left to evaporate slowly at room temperature. After one week, colorless needle crystals of the title compound were isolated (yield; 0.53 g, 71.1%).

### Refinement

The highest peak and deepest hole in the final difference electron-density map were located 0.98 and 1.12 Å, respectively, from atom Hg1. H atoms were positioned geometrically, with C-H = 0.93 and 0.96 Å for aromatic and methyl H, respectively, and constrained to ride on their parent atoms, with U_iso_(H) = xU_eq_(C), where x = 1.2 for aromatic H and x = 1.5 for methyl H atoms.

### Crystal data

**Table:**

[Hg(NCS)_2_(C_26_H_20_N_2_)]	*F*(000) = 2624
*M**_r_* = 677.21	*D*_x_ = 1.729 Mg m^−^^3^
Orthorhombic, *P**c**a**n*	Mo *K*α radiation, λ = 0.71073 Å
Hall symbol: -P 2n 2c	Cell parameters from 1276 reflections
*a* = 7.5907 (3) Å	θ = 1.7–29.3°
*b* = 24.0254 (10) Å	µ = 6.10 mm^−^^1^
*c* = 28.5284 (14) Å	*T* = 298 K
*V* = 5202.7 (4) Å^3^	Needle, colorless
*Z* = 8	0.40 × 0.05 × 0.04 mm

### Data collection

**Table:**

Bruker SMART CCD area-detector diffractometer	7051 independent reflections
Radiation source: fine-focus sealed tube	4018 reflections with *I* > 2σ(*I*)
graphite	*R*_int_ = 0.091
φ and ω scans	θ_max_ = 29.3°, θ_min_ = 1.7°
Absorption correction: multi-scan (*SADABS*; Bruker, 1998)	*h* = −10→10
*T*_min_ = 0.711, *T*_max_ = 0.789	*k* = −32→32
56419 measured reflections	*l* = −38→39

### Refinement

**Table:**

Refinement on *F*^2^	Primary atom site location: structure-invariant direct methods
Least-squares matrix: full	Secondary atom site location: difference Fourier map
*R*[*F*^2^ > 2σ(*F*^2^)] = 0.093	Hydrogen site location: inferred from neighbouring sites
*wR*(*F*^2^) = 0.199	H-atom parameters constrained
*S* = 1.21	*w* = 1/[σ^2^(*F*_o_^2^) + (0.0612*P*)^2^ + 12.3403*P*] where *P* = (*F*_o_^2^ + 2*F*_c_^2^)/3
7051 reflections	(Δ/σ)_max_ = 0.007
318 parameters	Δρ_max_ = 2.55 e Å^−^^3^
0 restraints	Δρ_min_ = −1.42 e Å^−^^3^

### Special details

**Table:**

Geometry. All e.s.d.'s (except the e.s.d. in the dihedral angle between two l.s. planes) are estimated using the full covariance matrix. The cell e.s.d.'s are taken into account individually in the estimation of e.s.d.'s in distances, angles and torsion angles; correlations between e.s.d.'s in cell parameters are only used when they are defined by crystal symmetry. An approximate (isotropic) treatment of cell e.s.d.'s is used for estimating e.s.d.'s involving l.s. planes.
Refinement. Refinement of *F*^2^ against ALL reflections. The weighted *R*-factor *wR* and goodness of fit *S* are based on *F*^2^, conventional *R*-factors *R* are based on *F*, with *F* set to zero for negative *F*^2^. The threshold expression of *F*^2^ > σ(*F*^2^) is used only for calculating *R*-factors(gt) *etc*. and is not relevant to the choice of reflections for refinement. *R*-factors based on *F*^2^ are statistically about twice as large as those based on *F*, and *R*- factors based on ALL data will be even larger.

### Fractional atomic coordinates and isotropic or equivalent isotropic displacement parameters (Å^2^)

**Table:**

	*x*	*y*	*z*	*U*_iso_*/*U*_eq_	
Hg1	0.43243 (6)	0.550529 (15)	0.12578 (2)	0.06252 (17)	
S1	0.1392 (5)	0.51033 (16)	0.1124 (2)	0.092 (2)	
S2	0.6577 (4)	0.47883 (13)	0.13551 (15)	0.0792 (11)	
N1	0.4970 (11)	0.6275 (4)	0.0798 (4)	0.046 (2)	
N2	0.4592 (12)	0.6271 (4)	0.1742 (4)	0.050 (2)	
N3	−0.089 (2)	0.5979 (7)	0.1294 (11)	0.103 (11)	
N4	0.451 (2)	0.3831 (6)	0.1223 (9)	0.110 (8)	
C1	0.487 (2)	0.5700 (6)	0.0093 (6)	0.077 (4)	
H1A	0.5691	0.5442	0.0230	0.115*	
H1B	0.5113	0.5736	−0.0236	0.115*	
H1C	0.3694	0.5567	0.0137	0.115*	
C2	0.5071 (14)	0.6259 (5)	0.0326 (5)	0.054 (3)	
C3	0.5329 (15)	0.6751 (6)	0.0080 (5)	0.059 (3)	
H3	0.5317	0.6737	−0.0246	0.071*	
C4	0.5594 (12)	0.7250 (4)	0.0287 (4)	0.045 (2)	
C5	0.5748 (13)	0.7761 (5)	0.0006 (4)	0.051 (2)	
C6	0.6648 (16)	0.7735 (6)	−0.0429 (5)	0.070 (3)	
H6	0.7145	0.7403	−0.0531	0.084*	
C7	0.677 (2)	0.8214 (8)	−0.0698 (5)	0.092 (5)	
H7	0.7444	0.8206	−0.0970	0.110*	
C8	0.5951 (19)	0.8693 (7)	−0.0578 (6)	0.084 (5)	
H8	0.6002	0.8999	−0.0777	0.101*	
C9	0.5032 (19)	0.8728 (7)	−0.0158 (7)	0.086 (5)	
H9	0.4500	0.9060	−0.0068	0.103*	
C10	0.4923 (14)	0.8254 (5)	0.0126 (5)	0.056 (3)	
H10	0.4279	0.8272	0.0403	0.068*	
C11	0.5596 (11)	0.7257 (4)	0.0793 (4)	0.042 (2)	
C12	0.6003 (11)	0.7739 (4)	0.1072 (4)	0.042 (2)	
H12	0.6380	0.8062	0.0923	0.050*	
C13	0.5857 (11)	0.7738 (4)	0.1538 (4)	0.045 (2)	
H13	0.6155	0.8056	0.1706	0.053*	
C14	0.5246 (10)	0.7252 (4)	0.1785 (4)	0.037 (2)	
C15	0.4939 (10)	0.7233 (5)	0.2277 (4)	0.041 (2)	
C16	0.5052 (12)	0.7738 (5)	0.2579 (5)	0.053 (3)	
C17	0.4287 (15)	0.8247 (5)	0.2438 (5)	0.067 (3)	
H17	0.3757	0.8279	0.2145	0.080*	
C18	0.433 (2)	0.8701 (6)	0.2741 (7)	0.084 (5)	
H18	0.3774	0.9031	0.2659	0.101*	
C19	0.521 (3)	0.8660 (9)	0.3164 (7)	0.101 (7)	
H19	0.5305	0.8973	0.3355	0.121*	
C20	0.594 (2)	0.8167 (9)	0.3307 (6)	0.091 (5)	
H20	0.6493	0.8142	0.3597	0.109*	
C21	0.5848 (15)	0.7709 (7)	0.3017 (4)	0.072 (4)	
H21	0.6328	0.7373	0.3117	0.086*	
C22	0.4508 (15)	0.6733 (5)	0.2475 (4)	0.053 (3)	
H22	0.4337	0.6713	0.2797	0.064*	
C23	0.4319 (14)	0.6253 (4)	0.2205 (4)	0.053 (3)	
C24	0.388 (2)	0.5713 (6)	0.2421 (6)	0.084 (4)	
H24A	0.2707	0.5607	0.2335	0.127*	
H24B	0.3963	0.5744	0.2756	0.127*	
H24C	0.4697	0.5435	0.2313	0.127*	
C25	0.5027 (11)	0.6756 (4)	0.1536 (4)	0.037 (2)	
C26	0.5208 (11)	0.6759 (5)	0.1032 (4)	0.037 (2)	
C27	0.011 (2)	0.5638 (6)	0.1219 (9)	0.098 (6)	
C28	0.5282 (17)	0.4244 (5)	0.1283 (7)	0.077 (4)	

### Atomic displacement parameters (Å^2^)

**Table:**

	*U*^11^	*U*^22^	*U*^33^	*U*^12^	*U*^13^	*U*^23^
Hg1	0.0714 (3)	0.03722 (19)	0.0789 (3)	−0.00113 (17)	0.0051 (3)	0.0002 (2)
S1	0.079 (2)	0.0619 (19)	0.126 (7)	−0.0174 (17)	−0.003 (3)	−0.026 (3)
S2	0.0708 (19)	0.0547 (15)	0.112 (3)	0.0096 (13)	0.006 (2)	0.0001 (18)
N1	0.062 (5)	0.038 (5)	0.039 (6)	0.002 (3)	−0.007 (4)	−0.004 (4)
N2	0.055 (5)	0.036 (4)	0.059 (7)	0.006 (4)	0.012 (4)	0.008 (4)
N3	0.093 (10)	0.077 (9)	0.12 (3)	−0.007 (7)	−0.007 (14)	−0.034 (15)
N4	0.116 (11)	0.066 (8)	0.13 (2)	−0.012 (7)	0.009 (14)	−0.015 (12)
C1	0.125 (11)	0.048 (7)	0.057 (9)	−0.001 (6)	−0.014 (7)	−0.013 (6)
C2	0.056 (6)	0.056 (7)	0.049 (8)	0.007 (4)	−0.006 (5)	−0.010 (6)
C3	0.061 (7)	0.080 (9)	0.037 (7)	0.001 (5)	−0.023 (5)	−0.004 (6)
C4	0.028 (4)	0.062 (6)	0.045 (6)	−0.004 (4)	−0.014 (4)	0.002 (4)
C5	0.047 (5)	0.060 (6)	0.046 (6)	−0.015 (5)	−0.018 (5)	0.012 (5)
C6	0.069 (8)	0.095 (9)	0.047 (8)	0.000 (7)	−0.010 (6)	0.020 (6)
C7	0.075 (9)	0.142 (15)	0.058 (9)	−0.016 (10)	−0.010 (7)	0.036 (10)
C8	0.084 (10)	0.099 (11)	0.069 (10)	−0.026 (8)	−0.017 (8)	0.045 (9)
C9	0.090 (10)	0.071 (10)	0.096 (14)	−0.011 (6)	−0.021 (8)	0.038 (10)
C10	0.057 (6)	0.061 (7)	0.051 (8)	−0.012 (5)	−0.013 (5)	0.017 (6)
C11	0.026 (4)	0.044 (4)	0.055 (6)	−0.004 (4)	−0.008 (4)	−0.001 (4)
C12	0.035 (5)	0.043 (4)	0.047 (6)	−0.003 (3)	−0.004 (4)	0.007 (4)
C13	0.042 (5)	0.048 (5)	0.044 (6)	−0.009 (4)	−0.004 (4)	−0.004 (4)
C14	0.022 (4)	0.054 (6)	0.034 (6)	0.001 (3)	0.004 (3)	−0.001 (4)
C15	0.027 (4)	0.054 (6)	0.040 (6)	0.004 (3)	0.004 (3)	−0.004 (5)
C16	0.042 (5)	0.066 (8)	0.050 (8)	−0.007 (4)	0.014 (4)	−0.008 (6)
C17	0.054 (6)	0.061 (7)	0.085 (10)	−0.005 (5)	0.025 (7)	−0.017 (6)
C18	0.085 (9)	0.072 (9)	0.097 (13)	0.004 (7)	0.034 (9)	−0.025 (8)
C19	0.128 (14)	0.098 (13)	0.077 (13)	−0.047 (10)	0.045 (10)	−0.047 (11)
C20	0.096 (11)	0.127 (15)	0.051 (9)	−0.026 (10)	0.009 (7)	−0.037 (9)
C21	0.062 (7)	0.115 (11)	0.038 (7)	−0.011 (7)	0.002 (6)	−0.010 (6)
C22	0.063 (6)	0.055 (6)	0.041 (6)	0.003 (5)	0.017 (5)	0.007 (5)
C23	0.052 (6)	0.048 (5)	0.059 (8)	0.005 (5)	0.021 (6)	0.010 (5)
C24	0.115 (11)	0.063 (7)	0.075 (10)	0.005 (7)	0.030 (8)	0.029 (7)
C25	0.040 (5)	0.034 (5)	0.036 (7)	0.006 (3)	−0.003 (4)	0.000 (4)
C26	0.032 (4)	0.047 (6)	0.031 (5)	−0.004 (3)	−0.008 (3)	−0.005 (5)
C27	0.076 (8)	0.055 (7)	0.16 (2)	−0.011 (6)	−0.007 (10)	0.037 (13)
C28	0.084 (8)	0.055 (6)	0.093 (11)	0.002 (6)	0.044 (8)	0.020 (8)

### Geometric parameters (Å, °)

**Table:**

Hg1—N2	2.309 (10)	C13—H13	0.9300
Hg1—N1	2.320 (10)	C14—C25	1.397 (15)
Hg1—S2	2.443 (3)	C14—C15	1.425 (15)
Hg1—S1	2.456 (4)	C15—C22	1.366 (16)
C1—C2	1.505 (18)	C15—C16	1.491 (17)
C1—H1A	0.9600	C16—C21	1.390 (18)
C1—H1B	0.9600	C16—C17	1.412 (18)
C1—H1C	0.9600	C17—C18	1.391 (19)
C2—N1	1.349 (16)	C17—H17	0.9300
C2—C3	1.389 (19)	C18—C19	1.38 (3)
C3—C4	1.351 (17)	C18—H18	0.9300
C3—H3	0.9300	C19—C20	1.37 (3)
C4—C11	1.445 (15)	C19—H19	0.9300
C4—C5	1.470 (14)	C20—C21	1.38 (2)
C5—C10	1.385 (18)	C20—H20	0.9300
C5—C6	1.416 (18)	C21—H21	0.9300
C6—C7	1.387 (19)	C22—C23	1.395 (16)
C6—H6	0.9300	C22—H22	0.9300
C7—C8	1.35 (2)	C23—N2	1.337 (16)
C7—H7	0.9300	C23—C24	1.476 (15)
C8—C9	1.39 (3)	C24—H24A	0.9600
C8—H8	0.9300	C24—H24B	0.9600
C9—C10	1.401 (19)	C24—H24C	0.9600
C9—H9	0.9300	C25—N2	1.346 (13)
C10—H10	0.9300	C25—C26	1.446 (14)
C11—C26	1.408 (14)	C26—N1	1.353 (14)
C11—C12	1.438 (13)	C27—N3	1.14 (2)
C12—C13	1.334 (15)	C27—S1	1.635 (18)
C12—H12	0.9300	C28—N4	1.17 (2)
C13—C14	1.441 (14)	C28—S2	1.648 (15)
			
N2—Hg1—N1	71.7 (3)	C13—C12—C11	122.2 (9)
N2—Hg1—S2	115.6 (2)	C13—C12—H12	118.9
N1—Hg1—S2	118.6 (2)	C11—C12—H12	118.9
N2—Hg1—S1	119.1 (2)	C12—C13—C14	121.0 (9)
N1—Hg1—S1	114.7 (3)	C12—C13—H13	119.5
S2—Hg1—S1	112.00 (12)	C14—C13—H13	119.5
C27—S1—Hg1	101.8 (5)	C25—C14—C15	116.9 (10)
C28—S2—Hg1	97.3 (5)	C25—C14—C13	118.9 (10)
C2—N1—C26	120.6 (11)	C15—C14—C13	124.1 (10)
C2—N1—Hg1	123.7 (8)	C22—C15—C14	118.4 (10)
C26—N1—Hg1	115.7 (8)	C22—C15—C16	119.4 (11)
C23—N2—C25	119.7 (10)	C14—C15—C16	122.2 (11)
C23—N2—Hg1	123.5 (7)	C21—C16—C17	118.6 (13)
C25—N2—Hg1	116.7 (8)	C21—C16—C15	120.2 (12)
C2—C1—H1A	109.5	C17—C16—C15	121.2 (12)
C2—C1—H1B	109.5	C18—C17—C16	119.4 (15)
H1A—C1—H1B	109.5	C18—C17—H17	120.3
C2—C1—H1C	109.5	C16—C17—H17	120.3
H1A—C1—H1C	109.5	C19—C18—C17	120.0 (16)
H1B—C1—H1C	109.5	C19—C18—H18	120.0
N1—C2—C3	119.2 (12)	C17—C18—H18	120.0
N1—C2—C1	117.3 (13)	C20—C19—C18	121.0 (15)
C3—C2—C1	123.4 (13)	C20—C19—H19	119.5
C4—C3—C2	123.8 (12)	C18—C19—H19	119.5
C4—C3—H3	118.1	C19—C20—C21	119.6 (16)
C2—C3—H3	118.1	C19—C20—H20	120.2
C3—C4—C11	116.6 (10)	C21—C20—H20	120.2
C3—C4—C5	120.9 (11)	C20—C21—C16	121.4 (15)
C11—C4—C5	122.4 (9)	C20—C21—H21	119.3
C10—C5—C6	118.3 (11)	C16—C21—H21	119.3
C10—C5—C4	122.8 (11)	C15—C22—C23	121.5 (11)
C6—C5—C4	118.7 (11)	C15—C22—H22	119.3
C7—C6—C5	118.7 (14)	C23—C22—H22	119.3
C7—C6—H6	120.6	N2—C23—C22	120.3 (10)
C5—C6—H6	120.6	N2—C23—C24	118.4 (11)
C8—C7—C6	122.3 (15)	C22—C23—C24	121.3 (12)
C8—C7—H7	118.8	C23—C24—H24A	109.5
C6—C7—H7	118.8	C23—C24—H24B	109.5
C7—C8—C9	120.1 (14)	H24A—C24—H24B	109.5
C7—C8—H8	120.0	C23—C24—H24C	109.5
C9—C8—H8	120.0	H24A—C24—H24C	109.5
C8—C9—C10	118.7 (17)	H24B—C24—H24C	109.5
C8—C9—H9	120.6	N2—C25—C14	123.1 (11)
C10—C9—H9	120.6	N2—C25—C26	117.5 (11)
C5—C10—C9	121.7 (14)	C14—C25—C26	119.3 (11)
C5—C10—H10	119.2	N1—C26—C11	121.4 (11)
C9—C10—H10	119.2	N1—C26—C25	118.3 (11)
C26—C11—C12	117.5 (10)	C11—C26—C25	120.3 (11)
C26—C11—C4	118.2 (9)	N3—C27—S1	174.3 (16)
C12—C11—C4	124.2 (9)	N4—C28—S2	173.6 (13)
			
N1—C2—C3—C4	4.2 (17)	C13—C14—C25—N2	174.8 (8)
C1—C2—C3—C4	−176.6 (12)	C15—C14—C25—C26	176.0 (8)
C2—C3—C4—C11	−0.2 (16)	C13—C14—C25—C26	−7.5 (12)
C2—C3—C4—C5	−176.2 (10)	C12—C11—C26—N1	−173.9 (8)
C3—C4—C5—C10	136.4 (12)	C4—C11—C26—N1	5.0 (13)
C11—C4—C5—C10	−39.3 (14)	C12—C11—C26—C25	7.1 (12)
C3—C4—C5—C6	−37.8 (14)	C4—C11—C26—C25	−174.1 (8)
C11—C4—C5—C6	146.5 (10)	N2—C25—C26—N1	−1.4 (13)
C10—C5—C6—C7	4.6 (16)	C14—C25—C26—N1	−179.3 (8)
C4—C5—C6—C7	179.1 (11)	N2—C25—C26—C11	177.7 (8)
C5—C6—C7—C8	−5(2)	C14—C25—C26—C11	−0.2 (13)
C6—C7—C8—C9	4(2)	C3—C2—N1—C26	−3.5 (15)
C7—C8—C9—C10	−2(2)	C1—C2—N1—C26	177.2 (10)
C6—C5—C10—C9	−3.1 (16)	C3—C2—N1—Hg1	174.9 (7)
C4—C5—C10—C9	−177.3 (11)	C1—C2—N1—Hg1	−4.4 (13)
C8—C9—C10—C5	2(2)	C11—C26—N1—C2	−1.1 (14)
C3—C4—C11—C26	−4.2 (13)	C25—C26—N1—C2	178.0 (9)
C5—C4—C11—C26	171.7 (8)	C11—C26—N1—Hg1	−179.6 (6)
C3—C4—C11—C12	174.6 (9)	C25—C26—N1—Hg1	−0.6 (10)
C5—C4—C11—C12	−9.6 (14)	N2—Hg1—N1—C2	−177.1 (9)
C26—C11—C12—C13	−6.5 (13)	S2—Hg1—N1—C2	73.2 (8)
C4—C11—C12—C13	174.7 (9)	S1—Hg1—N1—C2	−62.8 (8)
C11—C12—C13—C14	−1.2 (14)	N2—Hg1—N1—C26	1.4 (6)
C12—C13—C14—C25	8.4 (13)	S2—Hg1—N1—C26	−108.3 (6)
C12—C13—C14—C15	−175.4 (9)	S1—Hg1—N1—C26	115.6 (6)
C25—C14—C15—C22	2.0 (12)	C22—C23—N2—C25	−1.0 (16)
C13—C14—C15—C22	−174.3 (9)	C24—C23—N2—C25	−178.7 (10)
C25—C14—C15—C16	−177.3 (8)	C22—C23—N2—Hg1	179.9 (8)
C13—C14—C15—C16	6.4 (13)	C24—C23—N2—Hg1	2.2 (15)
C22—C15—C16—C21	42.1 (14)	C14—C25—N2—C23	1.3 (14)
C14—C15—C16—C21	−138.7 (11)	C26—C25—N2—C23	−176.5 (9)
C22—C15—C16—C17	−134.9 (11)	C14—C25—N2—Hg1	−179.6 (6)
C14—C15—C16—C17	44.4 (13)	C26—C25—N2—Hg1	2.7 (11)
C21—C16—C17—C18	−0.7 (16)	N1—Hg1—N2—C23	177.0 (9)
C15—C16—C17—C18	176.3 (10)	S2—Hg1—N2—C23	−69.4 (9)
C16—C17—C18—C19	3.7 (19)	S1—Hg1—N2—C23	68.4 (9)
C17—C18—C19—C20	−4(2)	N1—Hg1—N2—C25	−2.1 (6)
C18—C19—C20—C21	2(2)	S2—Hg1—N2—C25	111.4 (7)
C19—C20—C21—C16	1(2)	S1—Hg1—N2—C25	−110.7 (7)
C17—C16—C21—C20	−1.7 (17)	N2—Hg1—S1—C27	25.5 (10)
C15—C16—C21—C20	−178.7 (11)	N1—Hg1—S1—C27	−56.4 (10)
C14—C15—C22—C23	−1.8 (15)	S2—Hg1—S1—C27	164.8 (9)
C16—C15—C22—C23	177.5 (10)	N2—Hg1—S2—C28	141.9 (7)
C15—C22—C23—N2	1.3 (17)	N1—Hg1—S2—C28	−135.9 (7)
C15—C22—C23—C24	178.9 (11)	S1—Hg1—S2—C28	1.1 (7)
C15—C14—C25—N2	−1.7 (12)		
